# O8.7. COGNITIVE SUBTYPES IN FIRST-EPISODE PSYCHOSIS AND ASSOCIATION TO TREATMENT RESPONSE

**DOI:** 10.1093/schbul/sby015.243

**Published:** 2018-04-01

**Authors:** Manuela Russo, Simone Ciufolini, Olesya Ajnakina, Tiago Reis Marques, Avi Reichenberg, Anthony David, Marta Di Forti, Carmine Pariante, Robin Murray, Paola Dazzan

**Affiliations:** 1 Institute of Psychiatry, Psychology and Neuroscience, King’s College London; 2 Icahn School of Medicine at Mount Sinai

## Abstract

**Background:**

Psychotic disorders are characterized by large heterogeneity in clinical presentation, response to treatment and cognitive functioning. Indeed, there is evidence of the presence of cognitive subgroups of patients across affective and non-affective psychosis. However, very little is known about these subgroups in first episode psychosis (FEP) and whether they can be informative about course of illness, particularly response to treatment. The aim of this study is to investigate the number and the pattern of cognitive clusters in FEP, their external validity and association with treatment response at 12-week and 1-year follow up.

**Methods:**

The sample was composed by a total of 212 participants including 105 FEP patients from the South London and Maudsley Foundation Trust and 107 Healthy Controls (HC). All participants underwent a comprehensive clinical and neurocognitive battery. Z-score [mean=0, and standard deviation (SD)=1] were created for the whole sample based on the neurocognitive performance of the HCs. Treatment response at 12-week and 1-year follow-up was used to explore potential utility of subtypes in predicting response to treatment. Hierarchical cluster analysis was carried out to determine the number of cognitive clusters in FEP patients. A series of analyses of variance were carried out to determine if FEP clusters differed among each other in relation to demographic and clinical characteristics, level of functioning and from the HC sample in term of cognitive performance. Logistic regression was used to explore whether cognitive clustering was predictive of treatment response at 12-week and 1-year FU.

**Results:**

Four cognitive clusters emerged: one with near normal cognition (42.9% of the FEP patients) with a general cognitive score of z=-0.20, one with selected cognitive deficits (14.3%) in the domains of verbal memory, processing speed and executive functions (general cognitive score of z=-0.55); and two severe deficit clusters consisting in one cluster with severe deficits (33.3%; general score of z=-1.48) and the other with a deeply compromised cognitive ability (9.55%; general cognitive score of z=-2.34). There were no significant differences between clusters in terms of clinical features at baseline (including diagnosis, positive and negative symptoms, medication), apart from the level of functioning that was significantly lower in the severely compromised cluster compared to the near normal cognition cluster.

It emerged that majority (about 68%) of the patients from the near normal cognition cluster were responsive to treatment, whilst the majority of the selective and severely impaired clusters did not respond to treatment at 12-week follow-up. There were no significant results with regard to treatment response at 1-year FU.

**Discussion:**

Distinct patterns of cognitive impairments exist within FEP that might be characterized by different response to treatment. Clinical presentation at the onset of the illness is not useful in predicting response to treatment later on in the course of the illness, while cognitive functioning might be a more valid indicator. Cognitive stratification could represent a promising way forward to elucidate pathophysiology of psychosis and to provide tailored interventions.

